# How can we reduce biomedical research’s carbon footprint?

**DOI:** 10.1371/journal.pbio.3002363

**Published:** 2023-11-13

**Authors:** Frank J. Kelly

**Affiliations:** MRC Centre for Environment & Health, Imperial College London, London, United Kingdom

## Abstract

It isn’t easy being green in biomedical research. This Perspective looks at what changes in practice can make a difference now, and what challenges will need to be overcome to truly make a difference in the long run.

## Teaser

Biomedical research is a significant contributor to the global carbon footprint. Practices are available that could make a difference; however, there are significant obstacles ahead, including a lack of specialist expertise in sustainable research practices.

The impacts of climate change are knocking at the door. Here in the United Kingdom, a heatwave in July 2022 set new records—maximum temperatures reached unprecedented levels across large parts of the country, with temperatures exceeding 40°C in some places—the first time this has ever occurred in the UK [1]. As a consequence, that summer was the sixth driest on record, leading to the highest annual number of wildfires ever recorded in the UK. Meanwhile, in recent years, even worse events have occurred across the globe, with major wildfires devastating large land masses in Australia, Asia, Europe, and both North and South America. As a consequence, attention is rapidly turning to the challenge of reaching net zero carbon emissions by 2050 and averting the most damaging impacts of climate change.

**Fig 1:**
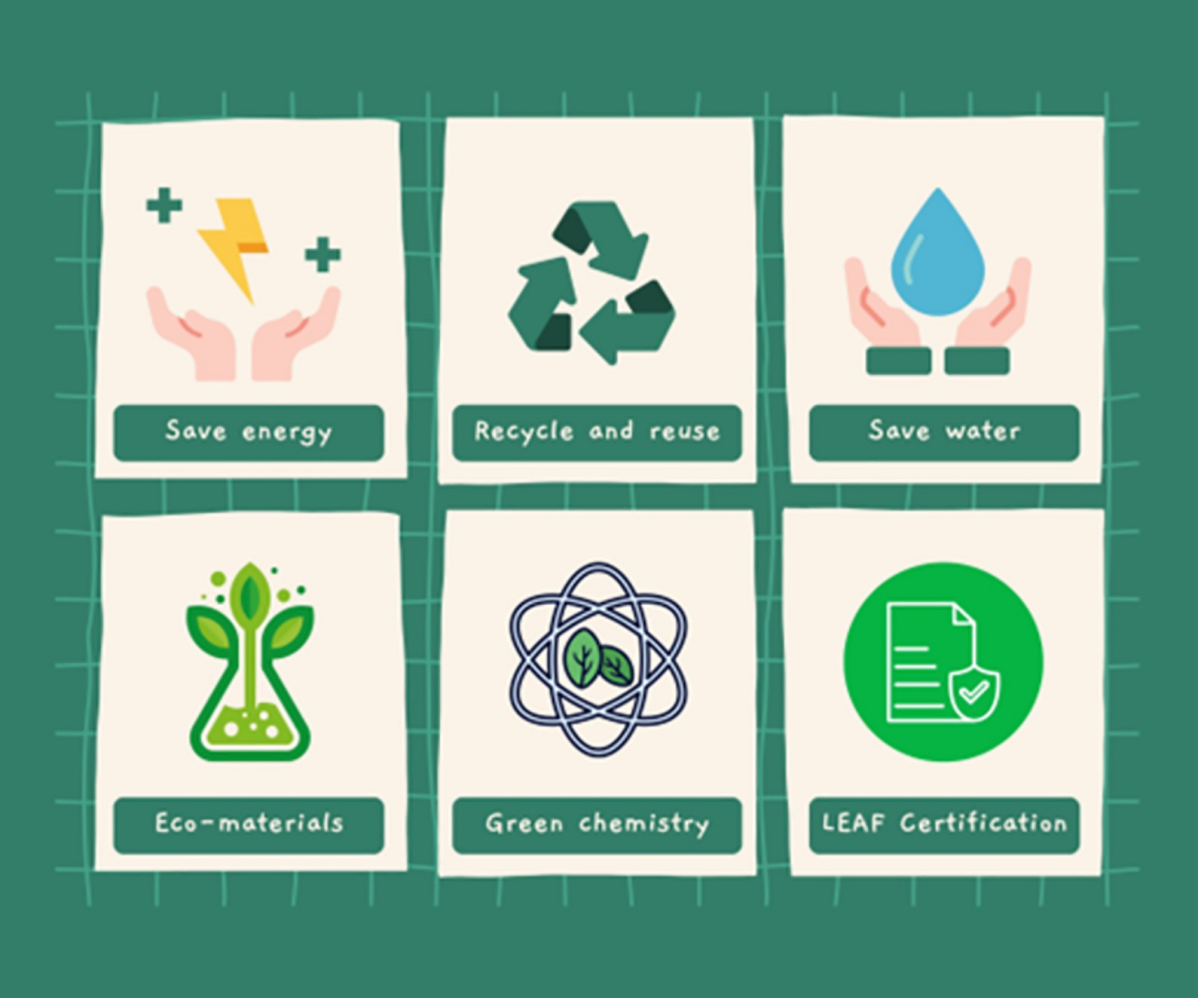
Sustainable laboratory practices. Save energy; recycle and reuse; save water; use eco-materials; utilise green chemistry; and get lab certification.

Laboratory research is a significant contributor to the carbon footprint of science, with the biomedical sciences having a particularly high contribution. For example, a −80°C freezer will use as much energy as a house and universities can have thousands of these [2]. Laboratories are resource intensive, using significant amounts of water and an average of 5 to 10 times more energy per m^2^ than standard office spaces [3], and bioscience research is responsible for almost 2% of global plastic waste [4]. Given that research and development investment in the UK is projected to increase by £80 billion over the next 10 years [5], to achieve the UK Government’s target of reaching net zero carbon emissions by 2050, it will be essential to minimise the carbon footprint of biomedical research here and elsewhere [4]. A good example of how support has developed in this area is the Laboratory Efficiency Assessment Framework (LEAF) program, which provides calculators and other practical resources and training for laboratory personnel on how to reduce their carbon footprint [2] (Fig 1). In one LEAF pilot project, an organisation was able to save 2.9 tonnes of carbon dioxide equivalent (tCO2e) and £3,700 across the 2 years. In addition to the environmental benefits, and health co-benefits, of environmentally responsible research, environmental practices can be cost-effective; for example, the reuse of labware can reduce the carbon footprint and running costs of laboratories.

In March 2023, as part of its FORUM programme of events bringing together representatives from the academic, industry, and health service sectors, the UK Academy of Medical Sciences co-hosted a 2-day workshop with the UK Medical Research Council and the UK National Institute of Health and Care Research to explore current initiatives to make biomedical research more environmentally sustainable and to identify potential next steps. The clear message that emerged was that there is a need to prioritise environmental sustainability within the biomedical research ecosystem [6].

Multiple pathways exist for improving the sustainability of biomedical research, such as improvements across the built environment, supply chains, and travel and transport, all of which would lead to significant emission reductions. Furthermore, green practices and carbon reduction targets have not yet been sufficiently embedded into the research system to make a difference. To move towards sustainability in biomedical research, several areas need to be prioritized:

- Top-down support for bottom-up activities and initiatives is essential. Much activity to date has been driven by “champions” with a commitment to sustainability. This bottom-up energy now needs to be matched by strategic commitments, investment, and action by key senior stakeholders in organisational leadership roles.
- We need to develop a research workforce (particularly including research support personnel) with specialist skills in green research practices. Research sustainability should be recognised as a specialist function in its own right and incorporated into job descriptions, with due attention given to issues such as capacity-building and career pathways.
- Sustainability efforts in scientific research should be coordinated, perhaps through a central entity, which could help develop common metrics and standards and bring together relevant information and case studies. Action is currently fragmented, with multiple individuals and bodies independently undertaking their own sustainability activities. Some efforts are being made to create consensus; for example, UK Research and Innovation (UKRI) is developing a concordat on sustainability to help align the activities of certain types of research organisations, such as funders and research institutions.
- Additional data and tools are needed to assist decision-makers (particularly researchers and project coordinators) to quantify, evaluate, and reduce environmental impact. Decision-making can be hampered by the fact that the carbon impact of many research activities cannot be accurately quantified, making it difficult for researchers, purchasers, and funders to make comparisons, prioritise actions, or determine the impact of interventions. More evidence about the environmental impact of research would therefore be useful to close key knowledge gaps and support decision-making processes.
- Training mechanisms need to be put in place so that researchers, students, and research support personnel can improve their sustainability awareness and actions. Individuals with experience and expertise in research practice and sustainability are needed to generate the evidence to support decision-making and promote good practice and to inform the development of guidance. There is a need to consider how best to nurture and grow this group of suitably trained professionals.

Above and beyond these priority areas, to prompt behaviour change to reduce environmental impact, messaging from funders and regulators should be clear. Presently, grant income is a highly valued metric in career progression, not efficient productivity. To change this perspective, greener research practice should be seen as an important part of good research practice, and incentives, rewards, and recognition for greener research practice should be introduced. Funding bodies should ensure that reporting on carbon emissions (and how they will be mitigated) becomes part of the requirement for funding bids. However, if funders were to consider the environmental impact of research during the grant application process, a standardised tool to assess a project’s environmental sustainability would be useful to allow comparability between applications. This, if instigated, would require universities to establish sustainability hubs to provide support much in the same way that finance and HR departments presently do. Given a fundamental change in how biomedical research is undertaken will require behaviour change and adjusted social norms in researchers at all levels.

A hub to support such conversations and the sharing of best practice is needed, and opportunities should be created for research support personnel and early career researchers.

We must be careful to minimise any potential tensions between increased sustainability and research priorities, in terms of speed and quantity of outputs. The concept of sustainability in research should be incorporated within existing expectations and requirements for research conduct, such as health and safety practices and ethical conduct [7]. By doing so, biomedical research can play its part in reducing the impact of climate change on the planet.
